# Effect of Gold Nanoparticle on 5-Fluorouracil-Induced Experimental Oral Mucositis in Hamsters

**DOI:** 10.3390/pharmaceutics12040304

**Published:** 2020-03-27

**Authors:** Carmem Jane Ferreira Vilar, Susana Barbosa Ribeiro, Aurigena Antunes de Araújo, Gerlane Coelho Bernardo Guerra, Raimundo Fernandes de Araújo Júnior, Gerly Anne de Castro Brito, Renata Ferreira Carvalho Leitão, Daniel de Lima Pontes, Luiz Henrique Da Silva Gasparotto, Maisie Mitchele Barbosa Oliveira, Anderson Dias Viana, Wendy Marina Toscano Queiroz de Medeiros, Breno Gustavo Porfírio Bezerra, Caroline Addison Carvalho Xavier de Medeiros

**Affiliations:** 1 Post Graduation Program in Biological Sciences, Federal University of Rio Grande do Norte, Avenida Senador Salgado Filho 3000, Lagoa Nova, Natal/RN 59078970, Brazil; carmemjaneferreiravilar@gmail.com; 2 Post Graduation Program in Biotechnology-RENORBIO, Federal University of Rio Grande do Norte, Avenida Senador Salgado Filho 3000, Lagoa Nova, Natal/RN 59078970, Brazil; susa05@gmail.com (S.B.R.); maisie.barbosa@gmail.com (M.M.B.O.); 3 Post Graduation Program in Dental Sciences/Post Graduation Program in Pharmaceutical Science/Department of Biophysics and Pharmacology, Federal University of Rio Grande do Norte, Avenida Senador Salgado Filho 3000, Lagoa Nova, Natal/RN 59078970, Brazil; auriprinino@gmail.com; 4 Post Graduation Program in Biological Sciences/Post Graduation Program in Pharmaceutical Science/Department of Biophysics and Pharmacology, Federal University of Rio Grande do Norte, Avenida Senador Salgado Filho 3000, Lagoa Nova, Natal/RN 59078970, Brazil; gerlaneguerra@gmail.com; 5 Post Graduation Program in Functional and Structural Biology/Post Graduation Program Health Science/Department of Morphology, Federal University of Rio Grande do Norte, Avenida Senador Salgado Filho 3000, Lagoa Nova, Natal/RN 59078970, Brazil; araujojr.morfologia@gmail.com; 6 Post Graduation Program in Morphofunctional Sciences/Department of Morphology, Faculty of Medicine, Federal University of Ceará, Rua Delmiro de Farias s/n, Rodolfo Teófilo, Fortaleza/CE 60416030, Brazil; gerlybrito@gmail.com (G.A.d.C.B.); renata.carvalho@ufc.br (R.F.C.L.); 7 Post Graduation Program of Chemistry/Institute of Chemistry, Federal University of Rio Grande do Norte, Avenida Senador Salgado Filho 3000, Lagoa Nova, Natal/RN 59078970, Brazil; pontesdl@quimica.ufrn.br (D.d.L.P.); lhgasparotto@gmail.com (L.H.D.S.G.); andersondiasviana@gmail.com (A.D.V.); wendymmedeiros@gmail.com (W.M.T.Q.d.M.); brenogpb@gmail.com (B.G.P.B.)

**Keywords:** mucositis, hamsters, 5-fluorouracil, NF-κB, gold nanoparticle

## Abstract

Background: Oral mucositis (OM) is a severe inflammation of the oral mucosal cells associated with chemotherapy and/or radiotherapy-induced toxicity, resulting in epithelial ulcers and higher risk of death from sepsis. The aim of the present study was to evaluate the nanoparticle (AuNp) effect on OM induced in hamsters. Materials and methods: 5-fluorouracil (5FU) was used on the first and second day of the experimental model in Golden sirian hamsters, and on the fourth day, mechanical trauma was applied to induce OM. The animals were divided into groups, i.e., polyvinylpyrrolidone (PVP), mechanical trauma (MT), 5FU, and groups treated with gold nanoparticles (AuNps) (62.5, 125, and 250 μg/kg). On the 10th day, animals were euthanized for macroscopic, histopathological, immunohistochemical, western blot, quantitative polymerase chain reaction (qRT-PCR), and AuNp quantification. Results: AuNp (250 μg/kg) reduced TNF-α, IL-1β, COX-2, NF-κB, TGF-β, and SMAD 2/3; increased glutathione levels; decreased the expression of Kelch ECH-associated protein 1 (KEAP1); and induced heme oxygenase 1 (*HMOX-1*) and NAD (P) H quinone oxidoreductase 1 (*NQO1*) genes. Conclusions: AuNp (250 μg/kg) prevented 5-FU-induced OM in hamsters and improved the parameters of inflammation and oxidative stress.

## 1. Introduction

Oral mucositis (OM) is characterized by acute and severe inflammation of the oral cavity [1]. This condition is associated with toxicity induced by chemotherapy and radiotherapy against epithelial cells of the oral mucosa and myelosuppression, which compromises the balance between cell proliferation and death, resulting in a thinner inflamed epithelium and the presence of ulcers. This causes pain and discomfort to the patient, and impairs the protective functionality of the oral mucosa [2]. The incidence of OM varies with the therapeutic regimen, and a higher incidence is reported in patients who associate chemotherapy with radiotherapy, reaching a percentage as high as 100% in individuals subjected to anticancer therapy for tumors of the head and neck [3]. During cancer treatment, patients with mucositis may present a percentage of bacterial and fungal infection twice as high as that in those without inflammation, which increases the risk of death due to sepsis, and therefore, the prevention of OM is essential for a good cancer prognosis. Despite its impact on patients, there are currently no effective treatment options to prevent or treat OM associated with chemoradiation therapy for cancer of the head and neck. Current clinical management of OM is largely focused on palliative measures, such as pain management, nutritional support, and maintenance of good oral hygiene, to prevent or reduce the severity of toxicity and to manage the associated symptoms, which will, in turn, enable the continued delivery of cancer therapy without interruption or dose reduction and improve the overall prognosis. In other words, the prevention of OM is crucial for a good cancer prognosis [3].

The use of anti-inflammatory drugs for the prevention and treatment of oral mucositis has been extensively described in the literature [4,5,6]. Nuclear factor kappa B (NF-κB) is the most studied classical pathway involved in the pathophysiology of OM. Research has shown that the gold nanoparticle (AuNp) has anti-inflammatory effects [7,8]. The use of agents that work by different mechanisms is a possible strategy for greater clinical effectiveness. Another approach that warrants further investigation is the use of novel drug delivery technologies. Nanotechnology has recently gained increased attention for its capability to improve bioavailability and therapeutic efficiency by increasing uptake of the active agent by target cells. Nanoparticles are used in nanomedicine as promising drug delivery systems, providing sustained or controlled drug delivery, improving the solubility of hydrophobic molecules, targeting drugs to specific tissues, and improving the drug profile, biodistribution, pharmacokinetics, and toxicity [9]. Nanocarriers are more effectively targeted to injured tissues, being justified by the diameter of the carriers and the increased permeability and retention capacity of inflamed tissues [10,11]. Passive targeting of NPs to inflamed tissues allows drug payload delivery to specific tissues and/or cells, potentially reducing side effects triggered by drug action in healthy tissues [12]. Gold nanoparticles (AuNp) have been shown to have anti-inflammatory effects. However, the effect of AuNp on OM, as far as we know, has not been described in the literature. AuNp blocks the activation of NF-κB by interacting with IKK-β and inhibits the production of proinflammatory cytokines, such as TNF-α and IL-1β [13,14,15].

In vitro studies have shown that AuNp elevates IL-10 cytokine levels, which partially explains its anti-inflammatory effect [13,14]. The literature also reports that AuNps may be involved in the regulation of the Kelch-like ECH-associated protein 1 (KEAP1)–nuclear factor E2-related factor 2 (NRF2) pathway, thus adjusting cytoprotective response to endogenous and exogenous stress caused by reactive oxygen species (ROS). At baseline, the factor NRF2, present in the cytoplasm, is inhibited by the KEAP1 protein. In the presence of ROS, KEAP1 is inactivated and releases the factor NRF2, which migrates to the nucleus, where it induces increased expression of heme oxygenase 1 (HMOX1), NAD (P) H quinone oxirreductase 1 (NQO1), glutathione, and superperoxide dismutase (SOD), resulting in antioxidant action.

In view of all this, gold nanoparticles represent a perspective for the treatment of inflammation and vectorization of drugs [15,16,17,18]. These particles are inert and easy to prepare; however, their effect on oral mucositis has not been investigated, as far as we know. Thus, the present study aimed to evaluate the effect of gold nanoparticle on oral mucositis induced by 5-fluorouracil (5-FU) in golden sirian hamster.

## 2. Materials and Methods

### 2.1. Synthesis of Gold Nanoparticles (AuNps) Stabilized by Polivynylpyrrolidone (PVP)

The production of AuNps was carried out according to a previously reported method [19]. Briefly, a glycerol/NaOH (Sigma-Aldrich, St. Louis, MO, USA) solution was added to AuCl_3_/PVP (Sigma-Aldrich, 99%; Sigma-Aldrich, 10,000) solution with magnetic stirring, to yield the following final concentrations: 1.0 mmol/L Au^3+^, 0.10 mol/L NaOH, 0.10 mol/L glycerol, and 10 g/L PVP. The final mixture had a deep-red color due to the formation of AuNps. The AuNps colloidal solution was allowed to rest for 16 h to ensure reaction completion. After adjusting the pH to 7.2, the gold nanoparticle solution was transferred onto a closed cellulose film and dialyzed for 2 h in deionized boiling water to purify the colloidal solution. The dialysis procedure was repeated 3 times. The nanoparticles were spherical in shape and had a mean diameter of 10 nm [20]. The final concentration of AuNPs was 100 μg/mL. A PVP solution of the same concentration as the AuNP solution was used as a control.

### 2.2. Experimental Induction of 5-Fluorouracil (5-FU) Oral Mucositis in Golden Sirian Hamster Groups

The study was approved by the Ethics Committee on Animal Use of the Federal University of Rio Grande do Norte (CEUA-UFRN) (protocol 071/2014, 28 December 2014). The experimental model of OM was induced in golden sirian hamsters (Mesocricetus auratus), males, with a mean weight of 160 g, maintained with a standard laboratory diet, water ad libitum, under a temperature of 22 ± 2 °C and light/dark cycles of 12 h [21]. Oral mucositis was induced by the intraperitoneal (IP) administration of 5-fluorouracil (5-FU) (Roche, Rio de Janeiro, Brazil) on the 1st and 2nd day, at doses of 60 and 40 mg/kg, respectively, followed by mechanical trauma. On day 4, under 10% xylazine (10 mg/kg, IP) and 10% ketamine (80 mg/kg, IP)-induced anesthesia, the right cheek pouch mucosa was irritated by superficial scratching to potentiate the oral mucositis, as previously described. The scratching comprised dragging the tip of a 22-gauge needle, twice in a linear manner, across the everted cheek pouch. The PVP or AuNp were given 30 min before chemotherapy (5-FU). The hamsters were euthanized with 2% thiopental (100 mg/kg) on the 10th day of the experimental model [22].

Oral mucositis is induced with 5-fluorouracil (5-FU) followed by mechanical trauma (MT) [5,22,23,24]. The induced mechanical trauma simulates irritating damages suffered by the oral mucosa of humans, which occur due to constant contact of the oral cavity with orthodontic appliances, maladaptive dental prostheses, and frequently used objects for hydration and feeding. These damages are promptly repaired in healthy individuals, in contrast to patients undergoing anticancer therapy [24].

The animals were divided into the following experimental groups (*n* = 5, per group): PVP Group: Animals without oral mucositis, PVP only was administered; group MT (mechanical trauma): Animals subjected only to mechanical trauma, only PVP was administered, animals without oral mucositis; group 5-FU (5-FU + MT + PVP): Animals subjected to experimental OM by the administration of 5-FU i.p. and mechanical trauma (MT), PVP was administered for 10 days, 30 min before chemotherapy (5-FU); and group AuNp (5-FU + MT + AuNp): Animals subjected to experimental OM by the administration of 5-FU IP and mechanical trauma (MT), different doses of gold nanoparticles (62.5, 125, or 250 μg/kg of AuNp, IP) was administered for 10 days, 30 min before chemotherapy (5-FU). Polivynylpyrrolidone (PVP) was used for the synthesis of gold nanoparticles (AuNps) and was used as a vehicle for animals not treated with gold nanoparticle: PVP, 5-FU, or MT groups [8].

The animals were euthanized on the 10th day of the experimental model, and the oral mucosae were analyzed macroscopically and extracted for histopathological analysis, cytokines and GSH (Reduced Glutathione) measurements, quantitative real-time polymerase chain reaction (qRT-PCR), immunohistochemistry, and quantification of gold nanoparticles in tissues.

### 2.3. Macroscopic and Histopathological Analyses

On day 10, the oral mucosa was exposed and clinically classified according to 6 score points: Score 0: Completely healthy mucosa, without erosion or vasodilation; score 1: The presence of erythema, with no evidence of mucosal erosion; score 2: Severe erythema, vasodilatation, and superficial erosion; score 3: Ulcer formation on one or more faces, affecting no more than 25% of the surface area of the mucosa, severe erythema, and vasodilation; score 4: Cumulative formation of ulcers, reaching about 50% of the surface area of the mucosa; and scale 5: Complete ulceration, making mucosal exposure impossible [5,25].

For histopathological analysis, the oral mucosa, liver, and lung were fixed in 10% buffered formaldehyde solution, dehydrated in alcohol baths (70% to 100%), diaphanized with xylol, impregnated, and included in a paraffin mold. The tissue blocks were cut with 5-μm microtome, fixed, and stained on histological slides with dewaxing in xylol, hydration in alcohol baths (100% to 70%), stained with hematoxylin and eosin, dehydrated in alcohol baths (70% to 100%), clarified with xylol, and slides were assembled for analysis by light microscopy (40× Nikon E200 LED, São Paulo, Brazil). Oral mucosa were classified into score: Score 1: Normal epithelium and connective tissue without vasodilation, absent or discrete cellular infiltration, or absence of hemorrhagic areas, ulcerations, or abscesses; score 2: Discrete areas of vasodilation or re-epithelialization, slight inflammatory infiltration with mononuclear prevalence, and absence of hemorrhagic areas, edema, ulcerations, or abscesses; score 3: Moderate vasodilation, areas of epithelial degeneration, inflammatory infiltration with neutrophil prevalence, presence of hemorrhagic areas, edema, and eventual ulceration, and absence of abscesses; and score 4: Severe vasodilation and inflammatory infiltrate with neutrophils [26].

Three sections of each organ (liver and lung) (*n* = 5) were analyzed in a double-blind fashion and evaluated semi-quantitatively according to the Ishak score [27]: 1—absence of fibrosis; 2—increase of the portal area; 3—fiber expansion in most portal areas; 4—presence of lobes with fibrous expansion in most portal areas with occasional portal bridges; 5—presence of lobes with fibrous expansion in most areas of the portal with a marked bridge (portal to portal and portal to central); 6—presence of a marked bridge (portal to portal and central portal) with occasional nodules (incomplete cirrhosis); and 7—presence of cirrhosis in the lobulos [7].

### 2.4. Assays for Quantification of Cytokines and Reduced Glutathione (GSH)

Cytokine quantification was developed using the enzyme-linked immunosorbent assay (ELISA) kit (R & D Systems, Minneapolis, MN, USA) [28]. In total, 100 mg of oral mucosa of each animal were homogenized with 600 µL of phosphate-buffered saline (PBS). The biological samples were homogenized in phosphate-buffered saline (PBS). Primary antibodies were incubated in Nunc microplates for 16 h at 4 °C, tween 20 was used to wash microplate wells, and they were blocked with bovine serum albumin (BSA). After standing and washing, the microplates were incubated with samples for TNF-α (detection range: 62.5–4000 pg/mL, sensitivity or LLD: 50 ng/mL of recombinant mouse TNF-α) and interleukin 1 β (2 h at 4 °C and detection antibodies for TNF-α detection range: 62.5 to 4000 pg/mL, sensitivity or LLD: 12.5 ng/mL of recombinant rat IL-1β). Remaining at rest under the same previous conditions, the microplates were stained with streptavidin, telltale solution (tetramethylbenzidine and hydrogen peroxide), and stop solution. The plates were read at 490 nm by an ELISA plate reader (Polaris, Celer, Belo Horizonte, Brazil). To obtain the results in pg/mL, the standard curve was obtained according to the manufacturer’s instructions for each antibody [24,26].

GSH was determined in the experimental groups (*n* = 5) by quantification of non-protein sulfhydryl radicals [29]. The samples were ground in a Politron Ultra-Turrax homogenizer with 1 mL of 0.02 M EDTA per 100 mg of tissue, and 400 μL of this homogenate was centrifuged at 3000 RPM along with 50% trichloroacetic acid, and 100 μL of the supernatant was transferred to the reading plate. The absorbance was determined at 420 nm immediately after the addition of 5,5-dithiobis (2-nitrobenzoic acid) (DTNB). The amount of non-protein sulfhydryl groups in the oral mucosa is expressed in mg/g tissue [6].

### 2.5. Histological Analysis by Immunohistochemistry

Immunohistochemistry was performed using the standardized method of streptoavidin-biotin-peroxidase. The mucosa tissue embedded in paraffin blocks were cut to a thickness of 3 μm on stoned silanized sheets (StarFrost^®^ Advanced Adhesive, Knittel, Braunschweig, Germany), which were dewaxed, rehydrated, with subsequent antigenic recovery by proteinase K. To block the endogenous peroxidase, 3% hydrogen peroxide was used for 10 min. Next, the slides were incubated for 18 h in a humidified chamber at 4 °C with the following primary polyclonal antibodies (Santa Cruz Biotechnology, Interprise, Brazil): Anti-COX-2 (1:400) and anti-NF-κB P65 (1:400). The secondary antibody was added (Biocare Medical, Concord, CA, USA) at room temperature to apply the horseradish peroxidase (HRP) conjugate (Biocare Medical, Concord, CA, USA). The immunoreactivity for various proteins was visualized after addition of DAB (3,3′Diaminobenzidine) chromogen against staining with Harry’s hematoxylin [7].

The colorimetric specimens were evaluated by an objective lens (400×) using optical microscopy (Planimetry microscopy-Nikon E200 LED), then scanned on the Pannoramic MIDI II scanner, and images were obtained using the Pannoramic Viewer software (3DHISTECH Ltd., Budapest, Hungary). The intensity of immunostaining was categorized as mild or intense by two examiners in a double-blind mode and classified into scores: Score 0: The absence of positive cells (0%); score 1: A small number of positive cells or isolated cells (<10%); score 2: Moderate number of positive cells (11%–50%); and score 3: A large number of positive cells (>50%) [30].

### 2.6. Western Blot for TGF-β and SMAD 2/3

For protein extraction, hamster oral mucosa samples (4 samples per group) were macerated in modified radioimmunoprecipitation lysis buffer (25 mmol/L Tris-HCl, pH 7.6; 150 mmol/L NaCl; 5 mmol/L EDTA; 1% NP40; 1% Triton X-100; 1% sodium deoxycholate; 0.1% SDS) with protease inhibitor and centrifuged (13,000 rpm, 17 min, 4 °C). After centrifugation, the supernatants were collected and the levels of total protein in each sample were measured by the bicinchoninic acid assay (Thermo Fisher Scientific, Waltham, MA, USA). In total, 40 µg of protein (previously prepared with sample buffer, Bio-Rad, and denatured at 95 °C for 5 min) were separated on sodium dodecyl sulfate–polyacrylamide gel electrophoresis (10%) under 120 V. The protein was then transferred to a polyvinylidene difluoride (PVDF) membrane (Bio-Rad) for 2 h under 100 V at 4 to 8 °C, blocked with 5% BSA (reconstituted in Tris-buffered saline (TBS) containing 0.05% Tween 20-TSBT) for 60 min at 4 to 8 °C, and incubated overnight with a primary antibody (mouse anti-β actin 1:500, Santa Cruz Biotechnology, SC81178; mouse anti-TGFβ 1/2 1:200, Santa Cruz Biotechnology, SC80346; rabbit anti-SMAD 2/3 1:400, Santa Cruz Biotechnology, SC11769) and a secondary antibody (goat anti-rabbit, 1:1000, Invitrogen, 656120; goat anti-mouse IgG, 1:500, Invitrogen, 626520;) for 90 min at room temperature. Then, the membranes were washed in TSBT, incubated in enhanced chemiluminescent (ECL, BioRad, Hercules, CA, USA) detection, and the image was captured with Chemi Doc MP (Bio-Rad). The Image J software (NIH, Bethesda, MD, USA) was used for densitometric quantification of the bands [31].

### 2.7. Real-Time Quantitative Polymerase Chain Reaction (qRT-PCR)

For the polymerase chain reaction, qRT-PCR assay, a mucosal homogenate was prepared with the Trizol reagent (Life Technologies, Carlsbad, CA, USA) to extract the ribonucleic acid (RNA). RNA was isolated with the SV Total RNA Isolation System kit (Promega Corporation, Madison, WI, USA). The purity of RNA present in the extracted volume was determined by the Nanodrop equipment (Thermo Scientific NanoDrop Products, Wilmington, DE, USA) [32]. The High-Capacity cDNA Reverse Transcription kit (Foster City, CA, USA) was used to convert the mRNA to complementary deoxyribonucleic acid (cDNA), by the action of reverse transcriptase with the programming of the thermal cycle for 10 min at 25 °C, 120 min at 37 °C, 5 min at 85 °C, and ∞ at 4 °C, to obtain a final volume of 20 μL of cDNA.

The oligonucleotide primers were designed using the Primer Express software version 3.0.1 (Applied Biosystems, Foster City, CA, USA). The following data was used to investigate the inhibitory activity of mesocricetus auratus, glycogendehyde 3-phosphate dehydrogenase (F: GAC TCA TGA CCA CAG TCC ATG C/R: AGA GGC AGG GAT GAT GTT CTG), heme oxygenase 1 (Hmox1) ACC TTC CCC AAC ATC GAC AA/R: TCA TGC GAG CGC GAT AGA G); NAD (P) H quinone dehydrogenase 1 (Nqo1) (F: GAA GCG CCT GGA GAC TGT CT/R: CAG GCT GCT TGG CAC AAA); kelch like ECH associated protein 1 (Keap1) (F: AGG GTC TCA CGT CTT CTC TTT GA/R: CCC CTT CTT CCC CTA GAA TTG A) (Applied Biosystems, USA).

The mRNA expression in real time was quantified by using the designed primers and the cDNA obtained. The Step One Plus thermocycler (Applied Biosystems, USA) was used for qRT-PCR in the assembled plate. To each well, 5 μL Power up syber green Master Mix, reverse (R) and forward (F) primers, and 2 μL of cDNA were added. The race was developed following the temperature cycle of 95 °C for 5 min followed by 40 cycles of 30 s at 95 °C, 52 cycles of 30 s at 60 °C (defined according to the primer), and a final cycle of 60 s at 72 °C. The specificity of the PCR products was confirmed by the melting curves. The comparative method Ct (cycle threshold) was applied to determine gene expression, C_t_ being the number of cycles required to produce the first fluorescence signal that exceeds the baseline, representing the beginning of the exponential amplification of the genetic material. This method analyzes the gene expression of the sample in relation to the control (PVP group), using the Ct values. Expression data were standardized using the reference gene *GADPH* in formula 2-ΔΔCt [5,33].

### 2.8. Digestion of Hepatic and Pulmonary Tissue by Microwave and Quantification of Gold Nanoparticles by Inductive-Coupled Plasma Optical Emission Spectrometry (ICP-OES)

Liver and lung samples from animals treated with a dose of 125 or 250 μg/kg of gold nanoparticles (AuNps) were used for the pre-digestion procedure in a MARS 6 microwave digestion system [34]. In the digestion tubes, 1.5 g of liver or 0.5 g of the lung with 12.8 mL of concentrated HNO_3_ (14.4 mol/L) and 3.2 mL of H_2_O_2_ were added. After a period of 12 h, the pre-digested samples were placed in a microwave oven under a constant power of 600 W for 20 min. The temperature varied throughout the process, reaching 177 °C to complete the digestion. The solutions obtained were cooled to room temperature, diluted to 25 mL with 2% HNO_3_, and stored appropriately for quantification of gold nanoparticles by inductive-coupled plasma optical emission spectrometry (ICP-OES).

The ICP-OES (iCAP 6300 Duo model, Thermo Fisher Scientific, Waltham, MA, USA) used for the determination of gold has axial and radial views, as well as a simultaneous charge injection device (CID) detector. Commercial 99.996% pure argon (White Martins-Praxair) was used [35]. The samples were pumped into the plasma with a peristaltic pump coupled to the equipment and its flow was controlled by the iTeva program of Thermo Scientific.

The analytical curve was prepared using a standard gold solution of 1000 mg/L in 10% nitric acid (AccuStandard Cat. No. ICP-22H-1, New Haven, CT, USA). We determined a blank and eight-point calibration with concentrations of 1, 2.5, 5, 10, 20, 40, 80, and 160 μg/L for the analytical curve, as well as for the quantification of the samples [34]. The calibration was externally performed with the stock solution of gold 100 µg/mL in 10% nitric acid.

The instrumental analysis parameters used in ICP-OES are listed in Table 1 [35,36]. To ensure adequate quality, measurement calibration spaces, checkpoints, and recalibration points were determined every 15 samples [36,37].

The gold element was quantified based on the linearity of the calibration curves, limit of detection (LD), limit of quantification (LQ), selectivity, accuracy, and precision, and validated to determine the gold element in liver and lung samples (Table 2) [38].

The gold element was quantified based on the linearity of the calibration curves, limit of detection (LD), limit of quantification (LQ), selectivity, accuracy, and precision, and validated to determine the gold element in liver and lung samples (Table 2) [38].

## 3. Results

### 3.1. Macroscopic and Histopathological Analysis of Oral Mucosa

**Figure 1:**
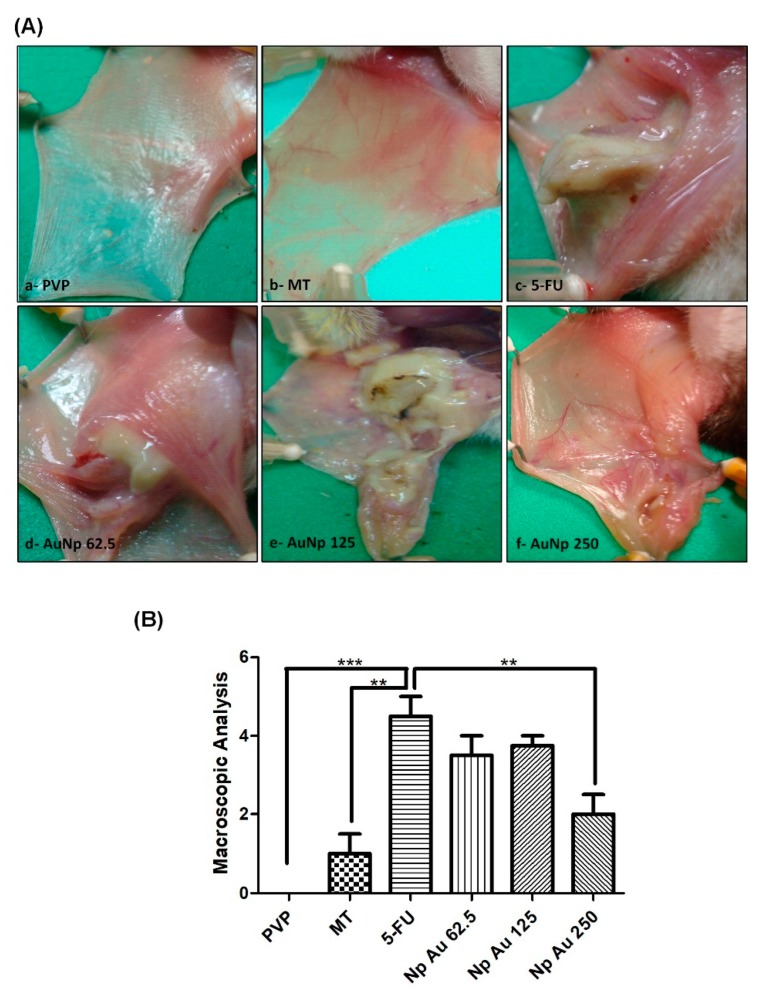
(**A**) Macroscopic analysis of the oral mucosa of hamsters (golden sirian) with oral mucositis (OM) induced by 5-fluorouracil (5-FU) and mechanical trauma (MT). PVP group: Animals with healthy, no erosion or vasodilatation of the oral mucosa, these hamsters only received PVP solution via i.p. (It is appropriate) or 10 days (**a**). Mechanical trauma (MT) group: Animals submitted to excoriation on the 10th day of the experimental model, the oral mucosa showed vasodilation and erythema (**b**). The 5-FU group: Animals that developed OM by administering 5-FU (IP) and mechanical trauma, the group showed oral mucositis characterized by ulcer formation, severe erythema, and vasodilation (**c**). The groups with OM treated with AuNp 62.5 (**d**), 125 (**e**), or 250 µg/kg (IP) (**f**). The animals treated with AuNp 250 µg/kg had mild vasodilation and superficial erosion in the oral mucosa, with no evidence of ulcers. (**B**) Score graph with the standard error of the mean (*n* = 5/group). ** *p* < 0.001; *** *p* < 0.0001 (Kruskal–Wallis test and Dunn’s multiple comparison test).

**Figure 2:**
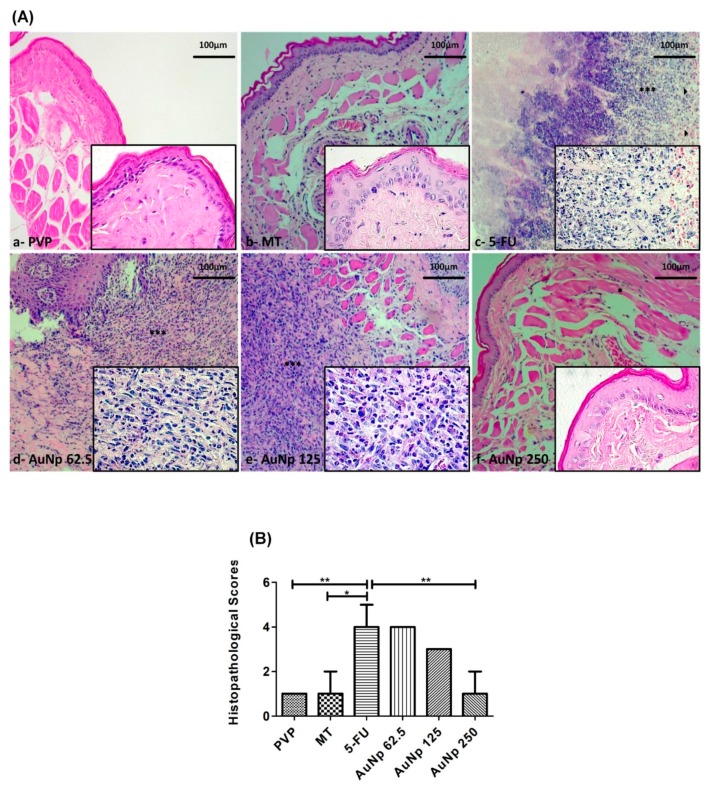
(**A**) Histopathological analysis of oral mucositis in hamsters on the 10th day of the experimental model. PVP group: Animals with normal tissue aspect (**a**). MT group: Animals without oral mucositis. (**b**). Group 5-FU: Animals with OM induced by 5-FU (**c**) and mechanical trauma, the oral mucosa showed areas of epithelial degeneration, hemorrhagic foci (►), intense inflammatory infiltrate (***), and regions suggestive of necrosis. Groups treated with AuNp 62.5 (**d**) or 125 µg/kg (**e**) did not improve oral mucositis. The group treated with AuNp 250 μg/kg (**f**) showed re-epithelialization with focal areas of inflammatory infiltration (*). (**B**) Score graph with the standard error of the median (*n* = 5/group). * *p* < 0.05; ** *p* < 0.001 (Kruskal–Wallis test and Dunn’s multiple comparison test).

The PVP group presented completely healthy oral mucosa epithelium, without erosion or vasodilatation, and the absence of pathological signs was observed both in the macroscopic aspect score 0 (0–0; *p* < 0.0001 vs. 5-FU) (Figure 1Aa,B) and in the histopathology score 1 (1–1; *p* < 0.001 vs. 5-FU) (Figure 2Aa,B). The MT presented slight alterations with the presence of erythema; however, no evidence of mucosal erosion, score 1 (0–1.5; *p* < 0.001 vs. 5-FU) (Figure 1Ab,B), fibroblasts, predominantly inflammatory infiltrate mononuclear, and vasodilatation in the histopathological analysis, score 1 (1–2; *p* < 0.05 vs. 5-FU) (Figure 2Ab,B). 5FU exhibited cumulative ulcer formation, reaching approximately 50% or even the entire surface area of the mucosa, making it impossible to expose it, score 4.5 (4–5; *p* < 0.0001 vs. PVP) (Figure 1Ac,B). Fibrinopurulent exudate, foci suggestive of necrosis, extensive ulcers, intense mononuclear inflammatory infiltrate, and hemorrhagic foci were also observed, score 4 (4–5; *p* < 0.05 vs. PVP) (Figure 2Ac,B). The groups treated with gold nanoparticles at the doses of 62.5 μg/kg, score 3.5 (1–4) (Figure 1Ad), score 4 (3–4) (Figure 2Ad), and 125 μg/kg, score 3.75 (1–4) (Figure 1Ae), score 3 (1–3) (Figure 2Ae), did not reverse OM changes induced by 5-fluorouracil; however, the dose of 250 μg/kg presented a lower macroscopic score than the group 5-FU, score 2 (1–2.5) (Figure 1Af). The oral mucosa of the group receiving 250 μg/kg of AuNp was coated with hyperceratinized, stratified squamous epithelium, with areas of hyperplasia, exocytosis, and underlying connective tissue, formed by randomly arranged collagen fibers with numerous fibroblasts and a mild to moderate inflammatory mononuclear infiltrate. Blood vessels completed the histopathological picture, score 1 (1–2; *p* < 0.05 vs. 5-FU) (Figure 2Af,B).

### 3.2. Histopathological Analysis of Liver and Lung and Quantification of the Gold Nanoparticle in Tissues

**Figure 3:**
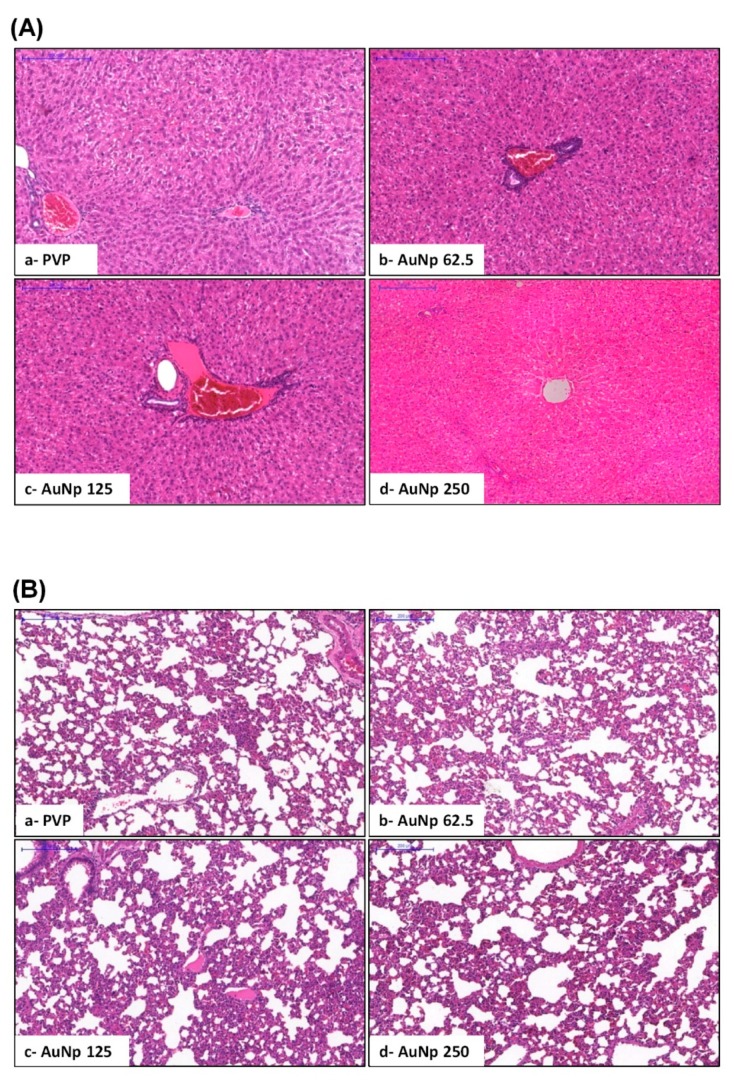
(**A**) Histopathological analysis of the hamster liver. (**B**) Histopathological analysis of the hamster lung. Both organs showed intact tissues with an aspect of normality. PVP group: Animals without oral mucositis (**a**). Groups with OM treated with AuNp 62.5 µg/kg (**b**); AuNp 125 µg/kg (**c**); AuNp 250 µg/kg (**d**).

Histopathological analyses of hepatic and pulmonary specimens of animals with oral mucositis treated for 10 days at doses of 125 and 250 μg/kg of AuNp showed no pathological alterations (Figure 3A,B). The hepatic transaminases, TGO and TGP, were within the normal range (data not shown), similar to the PVP group. Additionally, the quantification of gold in the tissues revealed that the concentration of this metal in the lung was below the detection limit, <0.0017 mg/L, indicating the low capacity of this organ to accumulate gold nanoparticles under the experimental conditions used. However, liver samples of animals treated with a 125 ug/kg dose of AuNp resulted in a concentration of 0.012 mg/L while for animals treated with 250 ug/kg, a higher level of gold was found as 0.063 mg/L

### 3.3. Quantification of Cytokines and Glutathione (GSH)

**Figure 4:**
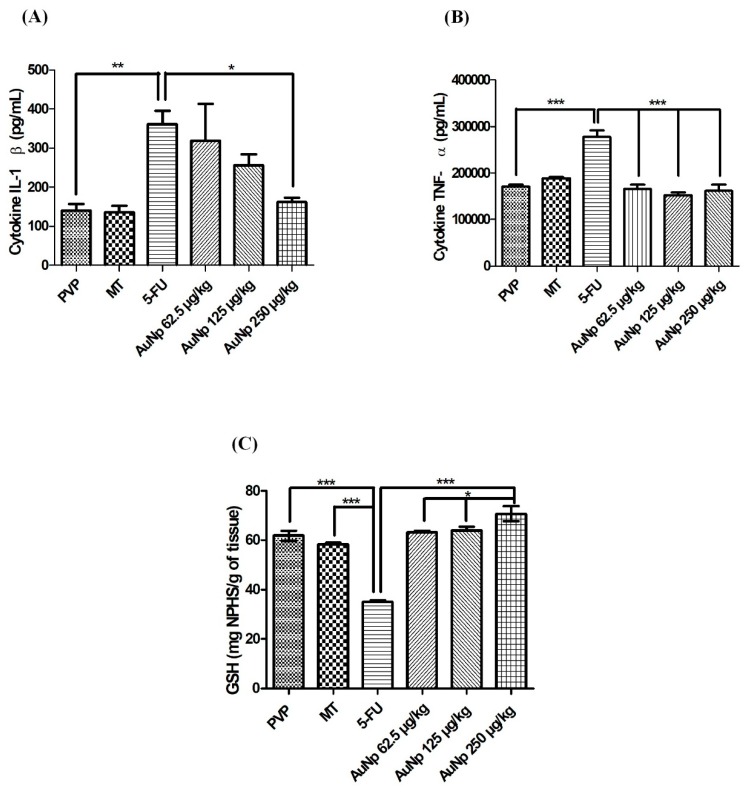
Quantification of interleukin 1 beta (IL)-1β (**A**), tumor necrosis factor alpha (TNF)-α (**B**), or glutathione (GSH) (**C**) in the oral mucosa on the 10th day of the experimental model of oral mucositis in hamsters. The PVP group: Animals without OM. The mechanical trauma (MT) group: Hamsters that received excoriations on the oral mucositis. The 5-FU group: Animals received 5-FU and were subjected to MT. The AuNp groups received 5-FU, were subjected to MT, and were treated with AuNp (IP) at one of three doses (62.5, 125, or 250 µg/kg). (*n* = 5/group). The results are presented as the mean ± standard error of the mean, (*n* = 5/group). * *p* < 0.05; ** *p* < 0.001; *** *p* < 0.0001 (analysis of variance with Tukey’s post-test).

The pro-inflammatory cytokines increased significantly in the animals with untreated oral mucositis (5-FU) compared to the PVP group: IL-1β (*p* < 0.001 vs. 5-FU) (Figure 4A) and TNF-α (*p* < 0.0001 vs. 5-FU) (Figure 4B). In contrast, the levels of these cytokines were attenuated in the groups with OM, treated with gold nanoparticles. AuNp at the dose of 250 μg/kg produced significant results in comparison to the 5FU group for IL-1β (*p* < 0.05 vs. 5-FU) (Figure 4A). The three doses of gold nanoparticles (62.5, 125, 250 μg/kg) decreased TNF-α levels in relation to the 5FU group (*p* < 0.0001 vs. 5-FU) (Figure 4B).

The animals with untreated oral mucositis presented a significant reduction of GSH in relation to the animals of the PVP and MT groups (*p* < 0.0001 vs. 5-FU) (Figure 4C). Treatment with the doses of 62.5, 125, and 250 μg/kg gold nanoparticles increased the GSH concentration compared to the 5-FU group (*p* < 0.0001 vs. 5-FU). A significant increase in GSH levels was observed in the group treated with 250 μg/kg of AuNp (*p* < 0.05 vs. AuNp 250 μg/kg) (Figure 4C).

### 3.4. Immunohistochemistry for COX-2 and NF-κB

**Figure 5:**
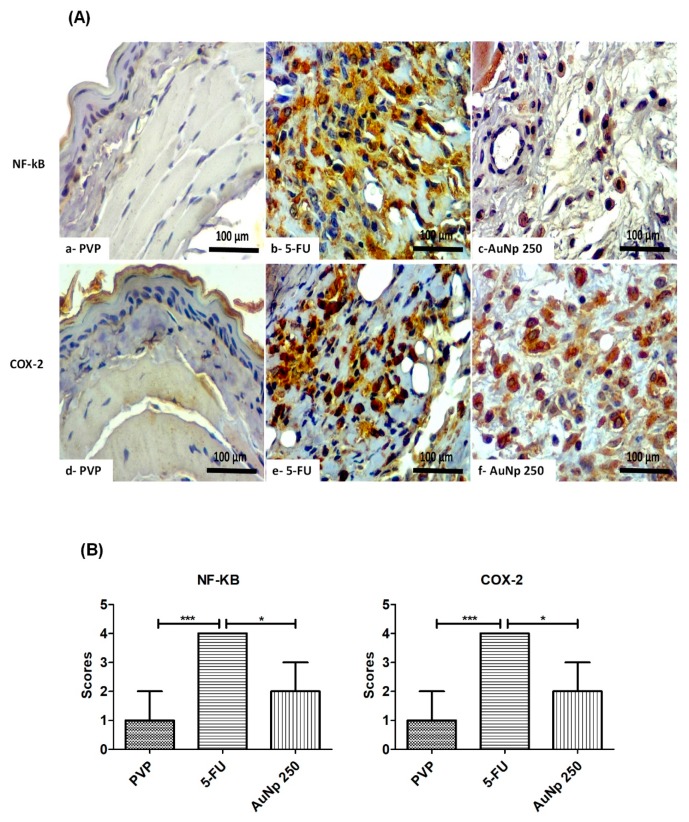
(**A**) Immunohistochemistry and (**B**) scores for NF-κB or COX-2. The PVP group with reduced immunostaining. The inflammatory cells of the oral mucosa in the 5-FU group had intense labeling for NF-κB or COX-2 compared to the PVP animals. In contrast, AuNp at 250 µg/kg reduced the immunostaining for NF-κB or COX-2, compared to the 5-FU group. (*n* = 5/group). * *p* < 0.05; *** *p* < 0.0001 (Kruskal–Wallis test and Dunn’s multiple comparison test).

Immunoexpression of NFκB in the PVP groups (score 1) was reduced as compared to 5FU (score 4). The gold nanoparticle dose of 250 μg/kg (score 2) markedly decreased the value compared to the 5FU group (* *p* < 0.05 vs. AuNp), the group with the highest immunoreactivity for NF-κB (Figure 5A,B). Similar findings were observed for COX-2 expression, which was significantly lower in groups with OM treated with AuNp 250 μg/kg (score 2) as compared to 5FU (score 4) (* *p* < 0.05 vs. AuNp). The PVP group (score 1) also showed reduced immunostaining, compared to 5FU (Figure 5A,B).

### 3.5. Western Blot for TGF-β and SMAD 2/3

**Figure 6:**
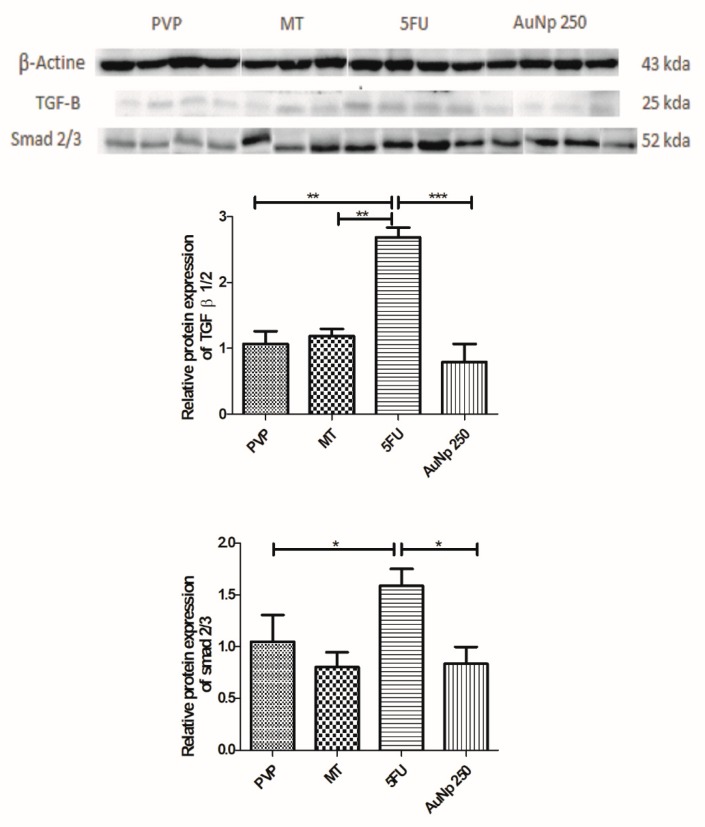
Representative images of TGF-β and SMAD 2/3 immunoblotting. The PVP group: Animals without OM. The mechanical trauma (MT) group: Hamsters that were subjected to mechanical trauma. The 5-FU group: Animals received 5-FU and were subjected to MT. The AuNp groups received 5-FU, were subjected to MT, and received AuNp at one of three doses (62.5, 125, or 250 µg/kg). The bands were visualized with an ECL system (BioRad). Lower expression of TGF-β or SMAD 2/3 was observed in the animals treated with AuNp 250 µg/kg as compared to the animals of group 5-FU. The chemiluminescence signal was detected with a ChemiDocTM XRS system (BioRad) and densitometrically quantified in ImageJ software (NIH, Bethesda, MD). The results are presented as the mean ± standard error of the mean, (*n* = 5/group). * *p* < 0.05; ** *p* < 0.001; *** *p* < 0.0001 (Analysis of variance followed by Tukey’s post-test.).

Expression of tumor growth factor β 1/2 (TFG-β 1/2) increased in the 5-FU group relative to the PVP group (** *p* < 0.001 vs. PVP). Animals with oral mucositis treated with gold nanoparticle (250 μg/kg) presented significantly lower levels compared to the 5FU group (*** *p* < 0.0001 vs. 5-FU). Treatment with AuNp (250 μg/kg) resulted in a reduction of SMAD 2/3 protein compared to the group with untreated oral mucositis (5-FU) (* *p* < 0.05 vs. 5FU) (Figure 6).

### 3.6. Quantification of Gene Expression by Quantitative Real-Time Polymerase Chain Reaction (qRT-PCR)

**Figure 7:**
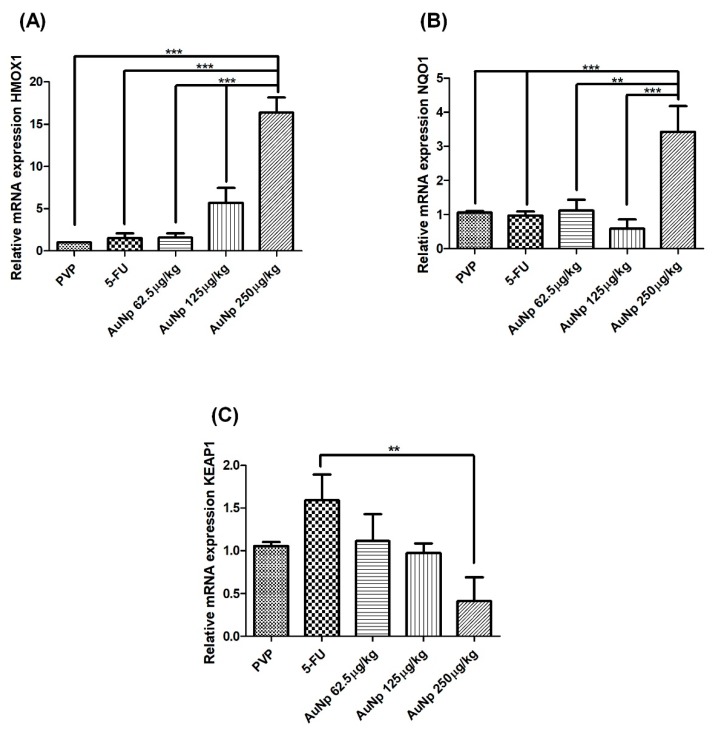
Real-time PCR for heme oxigenasse 1 (HMOX-1) (**A**); NAD(P)H quinone dehydrogenase 1 (NQO1) (**B**); and Kelch-like ECH-associated protein 1 (KEAP1) (**C**). The group AuNp 250 µg/kg increased the expression of the *HMOX1* or *NAD(P)H* genes compared to the 5-FU group. The AuNp 250 µg/kg decreased the KEAP1 mRNA expression compared to 5-FU animals. The results are presented as the mean ± standard error of the mean, (*n* = 5/group). ** *p* < 0.001; *** *p* < 0.0001 (analysis of variance with Tukey’s post-test).

Treatment of oral mucositis with AuNp (250 μg/kg) increased the gene expression of the antioxidant enzyme, hemeoxygenase 1 (HMOX-1), in relation to 5-FU animals (*p* < 0.0001 vs. 5-FU) (Figure 7A). Animals with oral mucositis treated with AuNp (250 μg/kg) had increased mRNA levels of the antioxidant enzyme NQO1 (NAD (P) H quinone dehydrogenase 1), compared to the 5-FU animals (*p* < 0.0001 vs. 5-FU) (Figure 7B). AuNp (250 μg/kg) reduced mRNA expression for the *KEAP1* (Kelch-like ECH-associated protein 1) gene (*p* < 0.001 vs. PVP and 5-FU) (Figure 7C).

### 3.7. Statistics

Data is presented as the mean of the group ± standard error. The analysis of variance (ANOVA) followed by the Turkey test was used to compare the mean values between the groups. Statistical analyses to compare macroscopic and histopathological scores were performed using standard group medians ± standard error, followed by the Kruskal–Wallis test and the Dunn multiple comparison test. Statistical analyses were performed on Prism 6 (GraphPad Software Inc., La Jolla, CA, USA), *p*-value < 0.05 indicated a statistically significant difference.

## 4. Discussion

In the present study, animals with untreated oral mucositis (5-FU) exhibited cumulative ulcer formation, reaching the entire surface area of the mucosa, with foci suggestive of necrosis, extensive ulcers, and intense inflammatory infiltrate. The gold nanoparticle (AuNp) at 250 μg/kg used once daily for 10 days reversed the clinical signs of OM observed in the macroscopic and histopathological results. The gold nanoparticles can easily enter the organs through the circulatory system, due to their tiny dimensions and high surface area for contact, inducing tissue damage due to oxidative stress [39]. Studies report that the diameter and concentration of gold nanoparticles used in living organisms determine whether the particle’s activity will be anti-inflammatory or inflammatory. In addition, the use of gold nanoparticles with sizes smaller than 10 nm and used at concentrations higher than 100,000 μg/kg (100 ppm) has higher inflammatory potential [40,41].

In this research, inductively coupled plasma optical emission spectrometry (ICP) demonstrated that animals with oral mucositis, treated with 125 and 250 μg/kg AuNp, had low levels of gold nanoparticles in hepatic tissue and levels below the level of detection in the lungs. The low levels corroborated the absence of histopathological alterations in the liver, supported by normal levels of ALT and AST transaminases (data not shown). Thus, in the current study, the 250 μg/kg AuNp dose reversed oral mucositis without causing toxicity in the liver and lungs. Studies have shown that the gold nanoparticle dose (AuNp 794.96 μg/kg) did not promote tissue changes [7], which supports our data. However, higher doses (AuNp 1500 μg/kg) caused damage to the lung tissue [20].

Our data show that treatment with gold nanoparticles at a dose of 250 μg/kg for 10 days significantly inhibits the production of proinflammatory cytokines, IL-1β and TNF-α. The proinflammatory cytokines, IL-1β and TNF-α, play an important role in the pathophysiology of oral mucositis. The production of these cytokines is induced by nuclear factor kappa B (NF-κB), a key pathway for the development of oral mucositis. NF-κB induces the expression of cyclooxygenase-2 (COX-2) and transforming growth factor beta (TGF-β) [5]. Authors have reported that AuNps reduce the levels of inflammatory markers in experimental models of periodontitis [42], 1% carrageenan-induced peritonitis [20], and ethanol-methamphetamine-induced hepatic injury [7], and positively influence the cellular response to infection or inflammation, interfering with the balance of cytokines involved in the inflammatory process [43].

Our analyses indicate that gold nanoparticles at the dose of 250 μg/kg reduce the immunostaining for NF-κB p65 and COX-2, and decrease TGF-β and SMAD 2/3 protein expression in relation to the 5FU group. Corroborating this data, authors have reported that NF-κB, COX-2, and TGF-β participate in the pathophysiology of OM [24]. The TGF-β is a superfamily of polypeptides and depending on the type of cell, it has negative or positive effects on cell proliferation and differentiation. In epithelial and endothelial cells, TGF-β disrupts the cell cycle, performing a pro-inflammatory action, delaying healing, and inducing apoptosis. TGF-β produced by keratinocytes and macrophages probably interacts with serine/threonine type II receptors, which phosphorylates the type I receptor by activating the SMAD 2/3 signaling pathway, resulting in the activation of proinflammatory factors. TGF-β can activate the NF-κB pathway, a classical pathway of the pathophysiology of oral mucositis [44].

In the present research, according to the methodology previously standardized by Gasparotto et al., 2012, the used nanoparticles exhibited neutral charge and pH 7.2. In addition, the size and the concentrations used showed anti-inflammatory activity. The nanoparticles increased GSH levels in the animals treated with a dose of 62.5, 125, and 250 μg/kg of gold nanoparticles. The best results were obtained with the dose of 250 μg/kg as compared to animals with untreated oral mucositis (5-FU). The literature shows that AuNp treatment induced anti-inflammatory and analgesic activity in an experimental model of paw edema in Wistar rats [8], as well as preventing cognitive damage and oxidative stress by increasing levels of GSH in a mouse model with Alzheimer’s disease [14]. Antioxidant systems are crucial for the body’s defense against oxidative cellular damage. GSH is one of the most abundant non-enzymatic antioxidants in all tissues of organisms, and it can eliminate free radicals, reduce peroxides, and be conjugated to electrophilic compounds through enzymatic or non-enzymatic reactions [45].

Research shows that AuNp may be involved in regulating the KEAP1 (Kelch ECH-associated protein 1)–NRF2 (nuclear factor erythroid 2-related factor 2) pathway, and adjusting the cytoprotective response to endogenous and exogenous stress caused by reactive oxygen species (ROS) [46]. Under baseline conditions, the KEAP1 repressor protein binds to the inactive NRF2 factor present in the cytoplasm. In the presence of ROS, KEAP1 releases the NRF2 factor that translocates to the nucleus, where it interacts with the antioxidant responsive element (ARE), inducing increased expression of heme oxygenase 1 (HMOX1), NAD (P) H quinone oxidoreductase 1 (NQO1), glutathione (GPx), and superperoxide dismutase (SOD), resulting in an antioxidant action [47]. Our results show that AuNp (250 μg/kg) attenuated mRNA expression for the *KEAP1* gene over 5FU animals. Furthermore, the reduction of KEAP1 mRNA expression suggests the release of the NRF2 factor with a consequent increase in the expression of HMOX-1 and NQO1 [48,49]. These data are consistent with our findings, where AuNp (250 μg/kg) increased GSH levels and gene expression of antioxidant enzymes, such as HMOX-1 and NQO1.

## 5. Conclusions

In this investigation, we demonstrated that AuNp prevented damage and inflammation induced by 5-fluorouracil in the oral mucosa of hamsters, most likely due to its anti-inflammatory and antioxidant effects. This data suggests that AuNps are promising for OM prevention and treatment. Father investigation must be conducted, however, to develop a safe and effective drug delivery system.

## Figures and Tables

**Table 1:** Instrumental parameters used for elemental determination by ICP-OES.

Parameter	Value
RF power	1150 W
Nebulizer gas rate	0.75 L/min
Auxiliary gas rate	0.5 L/min
Stabilization time	30 s
Display Mode	Axial
Replicates	3
Wavelength	Au (267.595 nm)

RF: Radiofrequency.

**Table 2:** Validation parameters.

Element	LOD (mg/L)	LOQ (mg/L)	Linearity	* Same Day	* Different Days
Au	0.0017	0.0051	0.9997440	1.0	4.5

* Mean of measurements of three aqueous samples containing gold element on the same day and on three different days.
